# Cholelithiasis in Infants: Risk Factors, Management, and the Role of Ursodeoxycholic Acid

**DOI:** 10.3390/children11121553

**Published:** 2024-12-21

**Authors:** Sevim Çakar, Gülin Eren, Cahit Barış Erdur, Mehmet Önder, Şafak Pelek, Sedef Alpdoğan, Duygu Demirtaş, Çiğdem Ömür Ecevit, Özlem Bekem

**Affiliations:** 1 Department of Pediatrics, Division of Pediatric Gastroenterology, Faculty of Medicine, Dokuz Eylül University, Izmir 35330, Turkey; 2 Department of Pediatrics, Division of Pediatric Gastroenterology, University of Health Sciences Dr. Behçet Uz Children’s Hospital, Izmir 35210, Turkey; gulin.eren@saglik.gov.tr (G.E.); cahitbaris.erdur2@saglik.gov.tr (C.B.E.); mehmet.onder2@saglik.gov.tr (M.Ö.); safak.pelek@saglik.gov.tr (Ş.P.); duygu.guner2@saglik.gov.tr (D.D.); cigdem.omurecevit@sbu.edu.tr (Ç.Ö.E.); ozlembekem.soylu@sbu.edu.tr (Ö.B.); 3 Department of Pediatrics, Faculty of Medicine, Ege University, Izmir 35040, Turkey; sedef.alpdogan@ege.edu.tr

**Keywords:** children, gallstones, infant, ursodeoxycholic acid

## Abstract

Background: Cholelithiasis is a rare disease in infants, and there is limited data on its risk factors and management. Objectives: To evaluate the risk factors, management, and response to medical treatment of cholelithiasis in infants. Methods: Infants diagnosed with cholelithiasis by ultrasound between 2018 and 2023 were retrospectively analyzed. Details of patient history, imaging findings, current symptoms, and treatments were reviewed. Results: Over 5 years, 98 infants were diagnosed with cholelithiasis. Thirty-three (33.7%) were girls, and the most common risk factors were the use of cephalosporin antibiotic therapy in 46.9%, sepsis in 30.6%, total parenteral nutrition in 29.6%, prematurity in 27.6%, congenital heart disease in 18.4%, and genetic disease (Down syndrome diagnosis in seven patients) in 16.3%. Only fifteen patients (15.3%) were symptomatic. Ursodeoxycholic acid (UDCA) treatment was given to 90.8% of patients, but nine of them used it for a short period or irregularly, and regular users were 81.6%. Gallstones disappeared in 46 patients (46.9%), including 14 (30.4%) without using UDCA regularly. The response rate to UDCA treatment was lower in preterm infants (*p* = 0.004). Gallstone resolution was higher in the nonusers, 14/18 (77.8%) versus 32/79 (40.5%) (*p* = 0.03). Acute cholecystitis was observed in only four patients; no other complications were noted. No infant required surgical or endoscopic treatment. Conclusions: UDCA should not be used routinely in children, especially infants, except in symptomatic children with a contraindication to surgery or to reduce clinical symptoms. In the absence of symptoms, patients may be monitored clinically.

## 1. Introduction

The incidence of cholelithiasis in children has been increasing over the years, although it is a rare disease, especially in infants. Management practice is heterogeneous. There is still a lack of guidelines on how to treat and manage gallstones and their complications in infants and children. Despite the increasing incidence in childhood, the current information is not sufficient. Moreover, gallstones are usually evaluated surgically in the literature. Many studies seem to agree on the association of delayed surgery with high complication rates, in contrast to the clinical practice in our hospital. Regarding the medical approach, Ursodeoxycholic acid (UDCA) treatment is recommended in asymptomatic patients according to updated German guidelines. However, in pediatric gastroenterologists’ practice, many symptomatic patients with cholelithiasis are under this treatment [1]. The ineffectiveness of UDCA in the treatment of cholelithiasis, other than symptomatic relief, has been reported in a small number of pediatric series [2,3]. Thus, we thought it would be useful to review infant gallstones from the gastroenterologist’s perspective. We aimed to evaluate the safe management of asymptomatic gallstones in infants, especially those who are followed up without any medical or surgical treatment.

## 2. Materials and Methods

This cross-sectional study was planned to evaluate data from a population of the tertiary care center, which included infants with cholelithiasis followed up in the Department of Pediatric Gastroenterology between 2018 and 2023. Patients aged 0–12 months with echographic evidence of cholelithiasis were included in the study. A diagnosis of cholelithiasis was based on the presence of echogenic foci producing acoustic shadowing in the gallbladder. Patients with a follow-up for less than one year were excluded from the study. None of these patients were using UDCA treatment before gallstones were detected. Age, gender, risk factors, symptoms, findings of ultrasound, medical and surgical treatments, and complications were retrospectively collected from the electronic medical records after the approval of the local ethics committee. In terms of clinical presentation, patients were categorized into two groups: asymptomatic and symptomatic. Symptomatic children were defined as having typical symptoms (emesis, jaundice, acholic stool, or right upper quadrant tenderness). Infants without gastrointestinal complaints who were diagnosed incidentally were defined as asymptomatic. If the gallstone disappeared completely or decreased in size by more than 5 mm at USG, UDCA treatment was considered to be effective.

### Statistical Analysis

The categorical data are given as a number of cases and percentages. The distribution of continuous data is evaluated by the Shapiro–Wilk test. The normally distributed variables are presented as mean and standard deviation (SD), while non-normally distributed variables are presented as median and interquartile range (IQR). The means and medians of the two independent groups were compared using the Student’s *t*-test and Mann–Whitney U test, respectively. The χ^2^ test or Fisher exact test was performed for the comparison of the categorical variables in different groups. The Statistical Package for the Social Sciences (SPSS) 22.0 (IBM Corp, Armonk, NY, USA) was used for all statistical analyses, and a *p*-value < 0.05 was considered statistically significant.

## 3. Results

Ninety-eight infants with gallstones who were 1 year of age or younger at diagnosis, including six who were <1 month old, were evaluated. The overall median age of diagnosis was 4.5 months (min 0-max 12 months), and 33.7% were girls. Twenty-seven (27.6%) patients had a history of preterm birth. Seventy-five (76.5%) children had one or more risk factors for gallstones (Table 1). The presence of cephalosporin use was the most common risk factor, with the highest rate of 46.9%.

Renal pathology was noted in five patients (nephrolithiasis in two patients, and renal Fanconi syndrome, type 4 renal tubular acidosis, and hydronephrosis in one patient each). One patient was diagnosed with methylmalonic acidemia, and another one was followed up for an unclear metabolic disorder. Down syndrome was diagnosed in seven patients, and Noonan syndrome was diagnosed in one patient. West syndrome was diagnosed in two patients, and Alexander disease, Smith-Magenis syndrome, and Alagille syndrome were diagnosed in one patient each.

One patient had a 7 mm dilatation of the common bile duct and is currently under clinical follow-up without surgery for a choledochal cyst. None had choledocholithiasis. Biliary sludge was found in 26.5% of patients, single gallstones in 35.7%, and multiple gallstones in 37.8%. Overall, the diameter of gallstones was smaller than 5 mm in 43.9% of patients, and in only two patients (2%), the diameter of gallstones was larger than 10 mm.

Eighty-seven percent of patients were asymptomatic. The remaining eleven (15.3%) were symptomatic, medically treated (UDCA, antibiotics, and hydration), and became asymptomatic in the follow-up. Four patients developed acute cholecystitis. They recovered with medical treatment and had no further complications. No children received surgical or endoscopic treatment. Complications were not observed in any case, and none of the asymptomatic patients became symptomatic during follow-up.

Ursodeoxycholic acid (UDCA) was given to 90.8% (*n* = 89) of patients, with 10.1% (*n* = 9) non-compliant with treatment. A total of 81.6% (*n* = 80) of patients received the lowest dose of UDCA of 10 mg/kg/day for at least 6 months. The resolution rate of gallstones was significantly higher (7/9, 77.8%) in patients who never took UDCA than in those under regular UDCA treatment for at least 6 months of minimum dose of 10 mg/kg/day (32/80, 40.0%) (*p* = 0.030). The response rate to UDCA treatment had no statistically significant association with age or gender. The median age of patients with response was 5.5 months (min 0, max 12 months), while the non-responsive group age was 4.0 months (min 0, max 11 months) (*p* = 0.322). The girls’ percentage in the UDCA responsive group was 37%, while in the non-responsive group, the percentage was 31.4% (*p* = 0.562). When the response rates of the patients with risk factors to UDCA treatment were evaluated, it was observed that the absence of response to UDCA treatment was significantly higher only in prematurity (*p* = 0.004) (Table 2). Gallstones disappeared in one patient with hemolytic disease who received UDCA treatment, and none of the three patients with anatomical variation responded. Resolution rates according to gallstone sizes after treatment were as follows: gallstone size in 73.9% was smaller than 5 mm, in 26.1% was 5–10 mm, and in 0.0% was bigger than 10 mm. There was no statistical significance between the rate of disappearance of gallstones and stone size after the use of UDCA (*p* = 0.228). No recurrence of stone formation was observed in any of our patients.

## 4. Discussion

As far as we know in the literature, our study is very valuable as it is the study with the largest number of patients in the infant age group diagnosed with gallstones. There is an increase in the number of patients diagnosed with gallstones, ranging from 1.9% to 4%, associated with widely available ultrasound and increased use of ceftriaxone and parenteral nutrition over the years, as well as premature births, which were also among the most common risk factors identified in our study [4]. Although the number of patients is increasing rapidly, the conservative approach is preferred over interventional surgical treatments, especially in the younger age group. A pediatric study from Canada with data from 20 years on cholecystectomies showed that the number of patients undergoing cholecystectomy increased over the years while the proportion of patients under 3 years of age decreased [5]. This data is supported by the fact that no interventional procedures have been performed on patients diagnosed at our center during these five years.

The rate of risk factors was higher at 76.5% compared to other pediatric studies [2,6]. However, the fact that the age groups of the patients included in these two studies were older and heterogeneous may be explained by the absence of common risk factors in infant age, such as age-related TPN intake and congenital heart disease. A study that included 58 infant-age group data reported that the presence of risk factors was 69%, which is closer to our data [7]. It is remarkable in this age group that the high rate of hemolytic disease in other studies (respectively 16.8% and 3%) was found only in 1.0% of our population [2,6]. The rate of patients with a family history was found to be higher at 11.2% compared to the rate of 2% in a study of 455 patients who had a cholecystectomy, while our rate is lower compared to another Italian pediatric study with a rate of 28.2% [2,6]. The lower rate of family history in risk factors in our study may be explained by the fact that these patients were younger, consumed less supplementary food, grew up in more closed environments, and had less exposure to the environment (such as water, food, and other environmental factors). Although the most common risk factor was ceftriaxone use, which is similar to the literature, a higher rate was found in our group compared to other studies with a 20% ratio [8]. The rational use of ceftriaxone may help to reduce the incidence of gallstones in this age group.

One of the studies demonstrated that younger age groups presented with a comparatively lower incidence of symptoms [9]. Two-thirds of children with cholelithiasis were symptomatic in another multicentre study [2]. This is a very high rate compared to the 15.3% symptomatic patients in our study population. It is a fact that older children are better at localizing pain than younger children, who tend to present with non-specific symptoms [10].

As a result of our data, we should also highlight that Down syndrome (DS) is one of the risk factors for gallstones, with a high rate of 7.1%. Patients with DS are at risk of developing cholelithiasis and should be monitored throughout the neonatal period, even if they have no other known risk factors for gallstone formation [11,12]. Many DS are asymptomatic, and gallstones resolve spontaneously in the majority of asymptomatic patients [12].

As only a small number of patients can benefit from UDCA as a dissolution therapy, its specific beneficial effect is related to the prevention of complications in symptomatic gallstones, which is independent of stone dissolution [13]. In uncomplicated cases, UDCA administration with close follow-up is recommended in Korean data [14]. The complete disappearance of small stones (<5 mm) in about 90% of patients after 6 months of UDCA treatment was reported in one clinical trial [15]. Conversely, a study that did not routinely recommend UDCA treatment suggests that clinically silent gallstones in children and infants have low complication rates and can be managed conservatively [7]. Another multicentre study supporting this view from Italy, including a surgical perspective, showed that gallstones persisted in 87.9% of those treated with UDCA [2]. These results are supported by our data, which shows that the rate of stone dissolution was almost twice as high in our patients who did not receive any treatment.

## 5. Limitations of the Study

It should be noted that this study is not without limitations, including its retrospective design and the relatively small sample size. In addition, some infants had more than one risk factor, such as cephalosporin use, sepsis, and TPN use, which may have contributed a confounding factor in determining a clear etiology.

## 6. Future Perspectives

The actual management of cholelithiasis in children is characterized by diagnostic and therapeutic heterogeneity, especially differences between pediatric surgeons and gastroenterologists. Establishing consensus between pediatric gastroenterologists and surgeons is the best way to approach symptomatic, asymptomatic, and complicated cholelithiasis and choose the best strategy [1,16]. In light of the absence of surgical complications, it can be posited that the lack of intervention during this period may be indicative of the feasibility of further observation and clinical follow-up. Prospective and controlled multicenter studies and international consensus conferences need to be organized to develop diagnostic and therapeutic consensus.

## 7. Conclusions

UDCA should not be used routinely in pediatric age, especially in infant age, except in symptomatic children with a contraindication to surgery or to reduce clinical symptoms. In the absence of symptoms, the patients may be monitored clinically and undergo serial abdominal ultrasound examinations.

Patients with DS are at risk of developing cholelithiasis. They should be monitored throughout the neonatal period, and in symptomatic and selected cases, medical or surgical treatment should be used.

## Figures and Tables

**Table 1:** Risk factors for the development of gallstones in infants.

Risk Factors	Number of Patients % 75/98 (76.5)
Ceftriaxone therapy	46(46.9)
Sepsis	30 (30.6)
Parenteral nutrition	29 (29.6)
Prematurity	27 (27.6)
Congenital heart disease	18 (18.4)
Genetic pathology	16 (16.3)
Dyslipidaemia	15 (15.3)
Family-history	11 (11.2)
Down syndrome	7 (7.1)
Surgery	6 (6.1)
Anatomical pathologies	3 (3.1)
Malabsorption	3 (3.1)
Hemolytic disorders	1(1.0)

**Table 2:** Disappearance or reduction rate of gallstones after Ursodeoxycholic acid (UDCA) treatment according to risk factors.

Risk Factor	Number of Patients Using UDCA	Number of Patients with Response %	Number of Patients with No-Response %	*p*
Ceftriaxone therapy	38	13 (34.2)	25 (65.8)	0.563
Sepsis	24	7 (29.2)	17 (70.8)	0.313
Parenteral nutrition	23	5 (21.7)	18 (78.3)	0.064
Prematurity	23	3 (13.0)	20 (87.0)	0.004
Congenital heart disease	16	5 (31.2)	11 (68.8)	0.561
Genetic pathology	14	5 (35.7)	9 (64.3)	0.879
Dyslipidaemia	13	5 (38.5)	8 (61.5)	0.761
Family-history	8	1 (12.5)	7 (87.5)	0.247
Down syndrome	6	2 (33.3)	4 (66.7)	NS
Surgery	6	2 (33.3)	4 (66.7)	0.826
Malabsorption	3	2 (66.7)	1 (33.3)	0.553

## Data Availability

The corresponding author will share the data upon request due to legal and ethical reasons.
